# ADVOCATING FOR AN AGE-INCLUSIVE WORKFORCE AND WORKSPACES: THE COLORADO EXPERIENCE

**DOI:** 10.1093/geroni/igae098.0410

**Published:** 2024-12-31

**Authors:** Jodi Waterhouse

**Affiliations:** CU Anschutz Multidisciplinary Center on Aging, Aurora, Colorado, United States

## Abstract

This session will focus on the advocacy work that is required at the state level to ensure that older adult worker rights are protected, both as they are looking for meaningful work with a livable wage or once in the workplace. We will describe a step-by-step process to advocate for and successfully implement policies to promote an age-inclusive workforce in Colorado – a state with one of the fastest-growing older adult populations and one of the most robust age-friendly community movements in the US. To help drive change within our state, we started by building community consortiums focused on education, advocacy, and sponsoring legislation to address identified workforce needs and opportunities. We will highlight the importance of a comprehensive needs assessment process in which community consortiums engaged as the basis for their advocacy work. Examples of consortiums, created by the Colorado Governor’s Office, will be shared as a model for symposium participants to create similar working groups to engage key constituents in their own states. We will also share our experience of crafting bills and seeing them through the legislative process, highlighting bills that we have sponsored and that have been passed in Colorado. Attendees will leave this session with more insights about how to work with their state’s elected officials to drive more support for an age-inclusive workforce and workplaces.

